# Modeling non-dual awareness *via* constraint closure: a reinterpretation of groundlessness

**DOI:** 10.1093/nc/niaf068

**Published:** 2026-01-20

**Authors:** Kiana Ward

**Affiliations:** Neuroscience Program, Amherst College, 25 East Drive, Amherst, MA 01002, United States

**Keywords:** constraint closure, emptiness, non-dual awareness, meditation, enactivism, consciousness

## Abstract

Non-dual awareness (NDA) refers to a shift in consciousness in which the usual distinction between subject and object dissolves, and experience is no longer structured by conceptual mediation or goal-directed regulation. Meling enactivist model describes NDA as a meditative disclosure of groundlessness—the recognition of emptiness (śūnyatā), that all phenomena lack intrinsic nature. While enactivism explains autonomy through process closure, this article argues that constraint closure, as developed by Nave, extends that framework by making explicit how autonomy is sustained through the continual regeneration of its own relational conditions. This refinement prevents process-closure models from being read in substantialist terms when applied to complex cognitive systems, where stability arises through ongoing transformation rather than fixed organization. Nave’s account builds on Juarrero theory of constraint causality, which replaces intrinsic forces with relational conditions—a view that parallels Nāgārjuna’s Madhyamaka analysis of dependent origination. Integrating these perspectives, I propose that NDA corresponds to a shift from decoupled to precarious constraints, revealing that cognition and awareness persist not through intrinsic foundations but through the dynamic regeneration of interdependent relations.

## Introduction

Traditional cognitive science conceives cognition as the internal processing of information about a pregiven world, guided by representations that mediate between an organism and its environment. This framework presupposes a fundamental separation between mind and world, in which meaning is imposed by a cognitive subject rather than enacted through interaction. Enactivism challenges this assumption by proposing that cognition arises through embodied self-organization: organisms sustain their own identity through continuous engagement with a dynamic environment (Varela 1979, Maturana and Varela 1980  Varela et al. 1991, Thompson 2007).

The biological notion of autopoiesis provides the paradigm for this principle. A living system, such as a cell, maintains its boundary and components through recursive processes that regenerate the very conditions of their continuation (Varela 1979, Maturana and Varela 1980 ). At this most basic level, meaning arises through the coordination of autonomy and adaptivity. Autonomy refers to a system’s capacity to produce and sustain itself as a distinct unity, while adaptivity denotes its ability to evaluate perturbations as beneficial or harmful to its continued existence (Di Paolo 2005, Barandiaran et al. 2009, Di Paolo et al. 2017). Together, these principles make possible sense-making—the organization of experience relative to an organism’s own norms of viability (Varela et al. 1991, Di Paolo et al. 2017).

A bacterium moving up a glucose gradient illustrates this organization. Glucose has no intrinsic property of “being food”; it becomes meaningful only relative to the bacterium’s metabolism. Its movement expresses the adaptive modulation of coupling that sustains viability. Meaning, therefore, is not detected but enacted through self-organizing activity that maintains the organism’s coherence. Enactivism generalizes this insight beyond biology, treating autonomy as a formal organizational principle in which a system’s activity continuously re-establishes the relations that constitute it as a distinct unity (Thompson 2007, Di Paolo and Thompson 2014).

At higher levels of organization, this self-producing capacity gives rise to cognitive autonomy—the agent’s ability to modulate its own sensorimotor coupling to maintain adaptive coherence under changing conditions (Froese and Di Paolo 2011). Subsequent developments extend this framework to the social and linguistic domains, showing that sense-making can also be participatory: meaning arises through relational dynamics that coordinate multiple autonomous agents (De Jaegher and Di Paolo 2007, Di Paolo et al. 2018). In this view, cognition is the ongoing enactment of meaningful relations through the reciprocal regulation of autonomy and adaptivity—an organism’s continual bringing-forth of a field of significance defined by its own viability conditions.

One phenomenological condition that tests the limits of cognitive-scientific frameworks for understanding the mind is non-dual awareness (NDA)—and enactivism is no exception. NDA refers to a state of consciousness in which the ordinary division between subject and object dissolves, and the sense of a separate self or ego temporarily falls away (Lutz et al. 2007, Josipovic 2014, Dunne 2015, Meling 2021). Experience unfolds without evaluative filtering, conceptual mediation, or goal-directed control, and awareness is no longer organized around a perceiving center. Within Buddhist contemplative traditions, such states are cultivated through systematic meditative training aimed at revealing the constructed nature of subject–object duality (Wallace 1999).

In Theravāda and Mahāyāna contexts alike, practices such as śamatha (stabilizing attention) and vipassanā (insight) refine the mind’s capacity for sustained observation, allowing habitual reification and evaluative grasping to subside (Wallace 1999). In Madhyamaka- and Yogācāra-influenced Mahāyāna traditions—especially the mahāmudrā and dzogchen lineages of Tibetan Buddhism—this process culminates in the direct recognition of awareness as non-dual, a reflexive knowing (rigpa) in which phenomena appear without being grasped as either internal or external (Garfield and Priest 2003). The dissolution of self-reference is not a trance-like loss of consciousness or mystical absorption but the unfabricated functioning of awareness once evaluative and representational scaffolds are relaxed (Lutz and Thompson 2003, Josipovic 2019).

Philosophically, this realization corresponds to Nāgārjuna’s Madhyamaka account of śūnyatā (emptiness) and pratītyasamutpāda (dependent origination): the insight that all phenomena—including self, mind, and world—arise only in dependence upon contingent conditions and therefore lack intrinsic essence (Nāgārjuna, Mūlamadhyamakakārikā, trans. Garfield 1995, Westerhoff 2009, Siderits and Katsura 2013). In NDA, this principle becomes experientially manifest: the mind directly apprehends its own groundlessness, revealing that coherence and intelligibility do not depend on any enduring substance but emerge from the dynamic interdependence of conditions.

In the Western scientific context, by contrast, consciousness is typically conceived as a hierarchical control system that predicts, evaluates, and regulates its environment through internal models (Friston 2010  Hohwy 2013, Clark 2016). In NDA, these predictive and self-referential functions temporarily subside, revealing a mode of cognition that remains coherent without invoking an internal subject or fixed point of control (Lutz and Thompson 2003, Josipovic 2014, Meling 2021). Because this form of awareness is largely incompatible with the assumptions of contemporary cognitive science, NDA provides a powerful empirical and theoretical test case for rethinking autonomy, consciousness, and self-organization in non-substantialist terms.

Building on this insight, Meling (2021) offers an enactivist interpretation of NDA as a transformation in the structure of sense-making itself. In ordinary cognition, the organism enacts meaning through the continual regulation of its coupling with the environment according to norms of viability—a mode Meling refers to as ‘phase-1 enaction’. In ‘phase-2 enaction’, these adaptive norms and evaluative orientations lose their organizing dominance. The system ceases to appraise experience in terms of self-preserving relevance and instead becomes aware of the contingent, relational basis of its own organization. Meling identifies this experiential recognition of contingency as groundlessness, corresponding phenomenologically to the dissolution of the subject–object polarity and philosophically to the Madhyamaka insight of emptiness (śūnyatā).

While Meling’s account captures the experiential transformation of NDA within the enactive paradigm, it remains grounded in process-closure models of autonomy, which describe systems as networks of mutually sustaining processes that maintain organizational identity. Process closure successfully represents metabolic and homeostatic forms of self-organization, where relatively stable process networks sustain viability over time. However, when extended to neural or cognitive systems, such models tend to understate the continual reorganization required for coherence, thereby risking an implicit assumption of structural invariance. In the context of NDA—where hierarchical regulation relaxes and coherence persists through transient interdependence—this assumption becomes especially problematic, as the notion of fixed process loops implicitly reintroduces the very substantialism that Madhyamaka and Buddhist phenomenology reject.

This tension raises a deeper problem for enactivist accounts of autonomy: if adaptivity is defined as the system’s capacity to evaluate and regulate its interactions according to norms of viability, how can a system remain alive or coherent once those evaluative norms are suspended, as in NDA? If autonomy depends on adaptivity, the apparent persistence of organization in NDA seems paradoxical. Meling’s framework identifies this suspension phenomenologically but leaves its organizational basis underspecified. From the standpoint of process closure, adaptivity’s relaxation would imply a loss of the very regulatory loops that sustain the system’s viability. Yet in NDA, coherence persists—not through norm-guided control, but through the continued operation of short-timescale processes that maintain minimal organization. Explaining this persistence requires a model of autonomy that can accommodate viability through instability—a task for which constraint closure is uniquely suited.

Kathryn Nave’s (2025) theory of constraint closure addresses this limitation by reframing autonomy not in terms of closed processes but as the continuous regeneration of constraints that shape those processes. Drawing explicitly on Alicia Juarrero’s (1999, 2023) theory of constraint causality, Nave extends Juarrero’s insight—that causal efficacy arises from relational constraints which delimit possibilities rather than impose forces—into a general dynamical framework for living and cognitive systems. In Nave’s account, constraints are inherently unstable: they must be constantly repaired, replaced, or reconstituted through the system’s own activity. Autonomy thus emerges not from the preservation of stable organizational patterns but from the ongoing negotiation of instability.

This shift aligns closely with Nāgārjuna’s conception of emptiness. Just as Juarrero denies that causes possess intrinsic powers independent of the relational structures that enable them, Nāgārjuna denies that phenomena possess intrinsic essence or self-nature (svabhāva). Both accounts understand coherence as arising through mutual conditioning rather than inherent existence. Nave’s framework translates this metaphysical insight into an organizational principle for living systems: a system persists not through fixed identity but through the continuous regeneration of the very constraints that make its organization possible.

This article follows a dialogical model of comparative philosophy: rather than reducing Buddhist insight to cognitive science or *vice versa*, it stages a reciprocal elucidation between Madhyamaka philosophy and enactivist systems theory. I take a dialogical, not assimilative, approach to cross-cultural cognitive science. Meling’s enactive model establishes how the experience of NDA can be understood as the loosening of adaptive regulation, but it does not yet specify how coherence is sustained once evaluative and representational scaffolds recede. Nave’s theory of constraint closure provides the organizational resources for this explanation. By shifting the focus from relatively stable networks of interdependent processes to the continual regeneration of relational constraints, constraint closure clarifies how a system can remain viable through ongoing reconfiguration rather than structural persistence. I therefore refine Meling’s enactive account of NDA by replacing a process-closure reading of autonomy with constraint closure: autonomy as the continual regeneration of the constraints that make organization possible. Practically, I (i) explain why NDA remains coherent when adaptive, decoupled regulation relaxes—modeling it as minimal closure dominated by precarious constraints—and (ii) situate this move within a dialogical, cross-cultural exchange where Nāgārjuna’s analysis of emptiness and Juarrero’s constraint causality illuminate, but do not collapse into, enactivist theory.

## Groundlessness in the enactive framework

In his 2021 article, Meling develops an enactive account of groundlessness by expanding upon Rosch’s (2017) introduction to the revised edition of ‘The Embodied Mind’. Within the enactive approach, cognition is not the internal representation of an objective world but the activity of sense-making: a relational process through which a living system enacts significance in interaction with its environment. Meaning is not discovered; it is brought forth from within the organization of the living system itself. As Meling writes, cognition is “a deeply relational embodied action through which a cognitive system enacts a world of significance” that is “not pregiven but brought forth from within the living cognitive system’s endogenous activity” (Meling 2021, p. 3).

Following Rosch, Meling terms this ordinary, norm-governed mode of engagement ‘phase 1 enaction’. Here the organism’s activity is organized by viability norms and sustained through regulatory feedback. Autonomy supplies the basis for a first-person perspective, and adaptivity allows that perspective to flexibly evaluate what is better or worse for survival. Cognition in this phase thus produces a coherent world structured around the system’s own norms and purposes. Multiple layers of regulation interact so that if one pattern of sense-making ceases, another can become dominant, preserving overall coherence.

Yet this very coherence conceals the deeper fact that neither “self” nor “world” possesses any intrinsic foundation. The meaningful world enacted through ‘phase 1’ sense-making is contingent and relational: its apparent stability is a functional achievement, not a metaphysical ground. Meling argues that the disclosure of this contingency—the recognition that the entire edifice of sense-making lacks inherent essence—constitutes the experience of groundlessness.

‘Phase 2 enaction’, corresponding to NDA, reveals this groundlessness directly. In this mode, the regulatory activity that sustains a subject–object distinction subsides. Cognition no longer orients toward the world through adaptive norms; instead, it becomes reflexively aware of its own arising as experience. Meling describes ‘phase 2 enaction’ as “an alternative mode of knowing that is neither based on an observer and an observed nor on an embodied enactment of norms,” through which “the mind can firsthand experience the groundlessness of the enacted edifice in which humans live” (p. 3).

Rosch (2017) similarly characterizes this awareness as one in which “the mind is neither absorbed nor separated but simply present and available'' Rosch (2017, p.xi). The mind does not interpret or judge but rests in a non-conceptual presence. As adaptive sense-making diminishes, what remains is not absence or void but direct, reflexive awareness—non-dual, self-luminous, and open to all phenomena without discrimination. Meling emphasizes that this is not an additional cognitive state achieved through effort but the unobscured condition of cognition when regulatory mediation falls away: “knowing groundlessness is phase 2 enaction knowing itself, unobscured” (p. 8).

This transition parallels the Buddhist notion of emptiness (śūnyatā). Varela et al. (1991) note that all phenomena “are free of any absolute ground and that such ‘groundlessness’ (śūnyatā) is the very fabric of dependent co-origination” (p. 144). From this perspective, cognition never uncovers a fixed external reality, nor is it anchored in a metaphysical subject; both self and world emerge co-dependently through interaction. The mind’s direct realization of this interdependence is what enactivism identifies as the experiential disclosure of groundlessness. Varela and colleagues liken it to “a reflection in a mirror—pure, brilliant, but with no additional reality apart from itself” (p. 225). This image captures the immediacy and non-reifying character of ‘phase 2 enaction’: cognition reflects itself without positing a substance behind appearance.

Meling’s account therefore reframes groundlessness not as metaphysical speculation but as a cognitive possibility latent within the enactive structure itself. When adaptive activity relaxes, the system’s dependence on contingent relational constraints becomes experientially transparent. This prepares the conceptual ground for interpreting NDA in terms of constraint dynamics rather than process networks. The following sections develop this shift by bringing Meling’s model into dialogue with Nāgārjuna’s critique of intrinsic causation and with contemporary theories of constraint causality and closure.

## Nagarjuna on emptiness and causality

Nāgārjuna’s Mūlamadhyamakakārikā (MMK) offers one of the most rigorous analyses of causation in the history of philosophy. Rather than rejecting causality, Nāgārjuna reveals that causal relations lack svabhāva—any intrinsic nature or self-existence. Every phenomenon arises only through dependent origination (pratītyasamutpāda): “When this is, that is; when this ceases, that ceases.” If cause and effect possessed independent essences, neither would be necessary to the other. Causation, therefore, is coherent only as coordination among interdependent conditions that have no fixed being of their own.

A familiar example helps clarify this relational point. We often say that a temperature below 0°C causes water to freeze. Yet this apparent sufficiency conceals further dependencies: pure water can remain liquid below 0°C unless microscopic impurities or disturbances provide nucleation sites for ice crystals to form. Neither “temperature” nor “water” possesses an intrinsic power to produce freezing; the transformation occurs only through the coordinated presence of multiple enabling conditions. Nāgārjuna’s account of pratītyasamutpāda anticipates precisely this structure of conditional dependence, in which what appears as a simple cause–effect relation dissolves into a network of co-arising factors. The freezing of water thus illustrates that causal necessity is never inherent but always contingent upon context—a physical analog of Nāgārjuna’s claim that all phenomena lack self-nature (svabhāva).

Nāgārjuna develops this argument through the tetralemma (catuṣkoṭi), which denies the four possible modes of intrinsic arising—(a) from itself, (b) from another, (c) from both, or (d) without cause. Each alternative presupposes a self-existent ground that dependent origination undermines. As Siderits and Katsura (2013, pp. 17–20) note, this is not a denial of causal relations but of any intrinsic link between independently existing entities. Their commentary on An Analysis of Conditions (MMK 1) shows that the impossibility of arising (utpāda) follows once the notion of self-nature (svabhāva) is abandoned. Westerhoff (2009, pp. 36–41) deepens this point by showing that even positing intrinsic nature contradicts the dependency structure required for explanation.

As Garfield (1995) observes, this dialectic is not nihilistic but relational: it shows that what appears as causal efficacy is simply the coordination of conditions without inherent source. In An Analysis of the Noble Truths (MMK 24), Nāgārjuna equates dependent origination and emptiness itself—"Whatever is dependently arisen, we declare that to be emptiness….” Emptiness is thus not a metaphysical substrate but the recognition that all phenomena, including causation, are conditionally constituted and conceptually designated (Westerhoff 2009, pp. 67–70, Siderits and Katsura 2013, pp. 277–78).

From a Madhyamaka perspective, the stability of the causal world is a pragmatic achievement rather than an ontological foundation. The world appears stable because relational patterns are continually maintained through co-dependent activity. Nāgārjuna’s analysis parallels the enactive claim that identity and meaning are not discovered but enacted through ongoing organization. Both frameworks reject any underlying substrate—substance, essence, or invariant structure—that grounds interaction.

Understanding causality as constraint rather than force brings this parallel into sharper focus. Nāgārjuna’s account of dependent origination anticipates the modern shift from efficient to constraint-based causation: effects arise through the limitation of possibilities within a network of co-arising conditions. This cross-traditional synthesis operates dialectically: Nāgārjuna’s critique of intrinsic causation destabilizes the substantialist assumptions of Western causal models, while constraint-based theories render Madhyamaka’s insight formally intelligible within systems theory. In this sense, Nāgārjuna’s reasoning is not mere negation but a dialectical logic of co-dependence—an insight that enactivism formalizes as reciprocal constraint. Each framework thus transforms the other, revealing a shared non-substantialist logic underlying both metaphysics and cognitive science.

## Constraint causality

Modern science inherited from classical mechanics a view of causation as efficient force transmission: causes produce effects through the transfer of energy or momentum across substances. This mechanistic picture, rooted in seventeenth-century physics, treats the world as a chain of temporally ordered pushes and pulls—linear, decomposable, and in principle reversible. While this model proved extraordinarily successful for describing idealized systems, it cannot account for the organized complexity of living, cognitive, or social phenomena. In such systems, behavior depends not merely on energetic interaction but on the constraints that shape which interactions are possible.

Alicia Juarrero’s (1999, 2023) theory of constraint causality redefines causation in precisely these terms. Instead of explaining change by reference to intrinsic powers, she argues that causes operate by restricting possibilities within a dynamical system. Constraints are not entities added to a system but relations among its processes—patterns of dependence, boundary conditions, and symmetries—that channel energy and matter without themselves supplying energetic force. When water flows through a pipe, the pipe does not “push” the water; it limits its trajectory. Likewise, in biological and cognitive systems, organization emerges not from external impulses but from the configuration of relational dependencies that make certain transformations viable and exclude others.

Juarrero situates this shift within a longer historical arc: from Aristotle’s formal and final causes, which described patterns and purposes, to the modern eclipse of form under efficient causation. She contends that recovering a notion of relational constraint allows contemporary science to reintegrate pattern and purpose without reintroducing teleology. Constraints are neither static essences nor external laws; they are emergent relations maintained through the system’s own dynamics. Their causal power lies in shaping trajectories rather than in transmitting force.

To articulate this in contemporary scientific language, Juarrero draws on complex-systems theory and the thermodynamics of open systems, where constraints define the region of state-space accessible to a system. Energy input is necessary but insufficient: only when energy flows are constrained does organization persist. For example, a pendulum’s motion depends on an initiating energetic impulse, yet the path it follows is determined by geometric and gravitational constraints. Without those constraints, energy would dissipate into random motion. Causation thus consists in the joint operation of energy and constraint—a view that resonates with Nāgārjuna’s rejection of intrinsic causal efficacy and with enactivism’s emphasis on relational organization.

Juarrero distinguishes two complementary levels of constraint:

1. Context-independent constraints, which specify lawful regularities (e.g. conservation principles); and
2. Context-dependent constraints, which arise from the system’s historical interactions and feedback processes.

This two-level model captures how higher-level organization both depends on and conditions lower-level dynamics—a circular relation absent from classical causation. Similar ideas appear in Montévil and Mossio (2015) and Moreno and Mossio (2015), who describe biological autonomy as closure of constraints: each constraint is maintained by processes that, in turn, depend on other constraints. Earlier precursors include Kauffman’s (1993) concept of work–task closure and Rosen’s model of closure to efficient causation. Juarrero’s contribution is to recast these formal insights in a dynamic, systems-theoretic vocabulary applicable to cognition and action.

At microscopic scales, constraint causality manifests in phenomena such as Brownian ratchets or molecular motors, where random thermal motion is rectified into directed work by asymmetric structures. The ratchet does not supply energy; it channels stochastic fluctuations, transforming disorder into usable organization. This exemplifies Juarrero’s point that constraint is not the negation of energy but its orchestration. The same logic extends at macroscopic scales. A city’s traffic flow, for instance, is not produced by any single vehicle’s motion but by the network of constraints—road layouts, traffic lights, and right-of-way conventions—that channel the collective behavior of drivers. These patterns do not exert force; they delimit possibilities, shaping global order without acting as local causes. In Juarrero’s terms, causal efficacy resides in the organization of relations that make some trajectories viable and others impossible. Just as the pipe channels water or the ratchet channels thermal noise, the city’s infrastructure channels motion through constraint rather than push or pull. At each scale, organization emerges from the coordination of limits rather than the transmission of force.

Philosophically, constraint causality dissolves the dualism between material mechanism and abstract law. Constraints are real but relational: they exist only through the interactions they modulate, and they have no ontological standing apart from the systems that instantiate them. This conception parallels Nāgārjuna’s analysis of dependent origination, in which causal relations exist conventionally yet lack intrinsic nature. Just as Nāgārjuna denies any underlying substance behind relational phenomena, Juarrero denies intrinsic essence behind causal efficacy. Both accounts treat causation as patterned dependence—an organization of possibilities rather than a transmission of force. In reframing causal power as emergent from relational structure, constraint causality replaces the mechanistic assumptions of classical physics with an ontology of interdependence. This framework provides the conceptual bridge to Kathryn Nave’s (2025) theory of constraint closure, which extends Juarrero’s model to explain how relational constraints sustain autonomy across timescales.

### From process closure to constraint closure

In the classical enactive model derived from autopoiesis, a system is autonomous insofar as its component processes recursively produce and maintain the network that in turn produces them—closure of processes in the sense articulated by Varela and colleagues (Varela et al. 1991). Nothing in this minimal criterion requires an invariant structure. The point is organizational: the processes form a circular domain of production that sustains the system’s identity.

The difficulty arises not as a contradiction but as an under-specification when we move from metabolic organization to neural and cognitive organization. In explanatory practice, process-closure accounts typically presuppose relative organizational stability at a given grain and timescale (e.g*.* a topology of recurrent interactions that is “the same enough” for the analysis to go through). At metabolic scales, this presupposition is usually harmless: the relevant cycles can be treated as quasi-stationary regimes. At neural and cognitive scales, however, coherence is achieved through continual reconfiguration across multiple timescales (plasticity, rapid recruitment and dissolution of assemblies, task-dependent coupling, etc*.*). If one relies only on process closure, the mechanism by which stability is maintained without tacit invariants remains opaque. The risk, then, is not that process closure posits an enduring essence, but that its applications can drift toward reifying pragmatic invariants to do explanatory work.

Nave (2025) articulates this limitation directly, observing that “process closure may be general enough to apply to all scales of biological organization, but it is also too general to distinguish living, intentional, or cognitive systems from the merely organized.” She further notes that “it is difficult to specify an invariant network of processes at a level both specific enough to individuate a particular organism and flexible enough to incorporate the possibility of such changes.” In other words, process closure alone lacks the resources to describe how living systems maintain identity through transformation—a gap that widens as organization becomes more plastic and history-dependent.

Constraint closure addresses precisely this gap while remaining continuous with the enactive program. It relocates the explanatory burden from what processes reproduce what to how systems continually regenerate the constraints that channel those processes. Constraints are metastable relational patterns (boundary conditions, couplings, symmetries, norms) that have no standing apart from the dynamics they modulate, yet must be actively repaired and re-established for organization to persist. On this view, autonomy does not depend on an invariant network; it depends on ongoing constraint regeneration across timescales. As Nave emphasizes, closure “does not mean stasis—it means a coherent circulation of constraint regeneration across timescales.”

### Orders of constraint

Constraint closure formalizes how systems sustain coherence through the regeneration of relations that operate across multiple temporal and organizational scales. Building on the closure tradition in theoretical biology—Rosen’s (1991) closure to efficient causation, Kauffman’s (1993) concept of work–task closure, and Moreno and Mossio’s (2015) distinction between constitutive and regulatory constraints—Nave (2025) extends this lineage into a unified dynamical account. Her key innovation lies in treating constraints not as fixed structural entities but as metastable relations that must be continuously reconstituted through the system’s own activity. Autonomy, in this view, is the organized persistence of constraint regeneration across timescales.

Nave distinguishes three general orders of constraint, each reflecting a different degree of temporal and functional decoupling from the system’s immediate energetic exchanges. At the most fundamental level lie precarious constraints, directly sustained by the throughput of energy and matter. These include metabolic cycles, membrane potentials, and molecular repair processes that collectively enable continued viability. Their stability is precarious in a thermodynamic sense: they exist far from equilibrium and must constantly counter entropic decay through work. In living systems, processes such as Adenosine Triphosphate (ATP) hydrolysis, ion transport, and macromolecular turnover perpetually restore these constraints against degradation. As Nave emphasizes, this continuous dissipation of free energy is not incidental but constitutive—autonomy requires the ongoing regeneration of the very asymmetries that allow further work to occur. Precarious constraints thus instantiate the minimal thermodynamic condition for life: an organized flux that maintains form without appealing to intrinsic stability. They are biophysical and organizational, not phenomenological; they ground the possibility of cognition but are not themselves cognitive, interoceptive, or affective in character.

From this energetic foundation emerge decoupled constraints, slower regulatory patterns that modulate how precarious constraints are configured without being immediately reducible to them. Examples include homeostatic and adaptive mechanisms such as gene regulation, neural plasticity, and hormonal feedback loops. These introduce historical flexibility by allowing the system to reorganize its constitutive dynamics in response to shifting conditions, thereby extending coherence across longer timescales.

At the highest level, double-decoupled constraints comprise representational and abstract structures—linguistic, conceptual, or normative—that can remain relatively stable even as the underlying biological or neural processes fluctuate. Such constraints depend on collective or symbolic maintenance rather than any single organism’s material continuity, illustrating how constraint closure generalizes beyond metabolism to cognition and culture.

Nave’s taxonomy refines the framework of Moreno and Mossio (2015) by emphasizing that each level arises through temporal decoupling: slower, globally integrated interactions depend upon, yet also reshape, the faster dynamics that sustain them. The system’s coherence results not from hierarchical layering but from reciprocal dependency—each order of constraint presupposes the viability of the one below even as it modifies its boundary conditions in return. Stability, therefore, is achieved not by static structure but by continuous cross-scale regeneration.

This recursive organization dissolves the residual dualism between process and structure that persists in process-closure models. Constraint closure shows that organization itself is a distributed activity—life and cognition are not maintained by enduring substrates but by the ceaseless renewal of relational conditions. By grounding autonomy in the thermodynamic regeneration of precarious constraints and their higher-order modulations, Nave offers a general model of living and cognitive systems as coherent precisely because they are historically contingent. This view prepares the conceptual ground for understanding NDA as a mode in which such regenerative coherence persists under conditions of maximal openness.

### Philosophical implications

Constraint closure removes the residual substantialism that can remain implicit in process-closure models—particularly when those models are extended from basic metabolic systems to complex neural and cognitive organizations. Process closure, as originally formulated, defines autonomy in terms of a closed network of self-producing processes that together constitute a system’s identity. Within metabolic domains, this formulation poses no issue: the relevant cycles can be treated as quasi-stationary and physically bounded. But at higher levels of organization, such as neural or cognitive dynamics, coherence arises not through static self-production but through continual reconfiguration across timescales. Assuming a stable organizational network in such cases risks treating dynamic coherence as if it were underwritten by an enduring form—an implicit substantialism that conflicts with both relational biology and enactive theory’s anti-essentialist commitments.

Nave’s model resolves this tension by redefining autonomy as the regeneration of constraints across timescales rather than as the maintenance of a fixed network. Because constraints are transient relations—patterns of coordination that exist only in and through their ongoing re-enactment—identity and coherence are achievements, not possessions. Nave emphasizes that autonomy “is not preserved through invariance, but through the continuous repair of the very relations that define viability.” On this view, stability is not a background condition but a local and temporary effect of successful constraint regeneration. The system’s “identity” is nothing over and above the transient pattern of this activity.

## Reframing non-dual awareness

Recapping the argument: process closure sets the minimal criterion for autonomy as the self-producing organization of processes; constraint causality explains efficacy as arising from relational conditions rather than intrinsic forces; and constraint closure specifies how those relations are actively renewed across scales. Nāgārjuna’s analysis of dependent origination, Juarrero’s theory of constraint causality, and Nave’s model of constraint closure converge on a shared insight: organization and efficacy arise not from essences or fixed substrates but from the ongoing coordination of interdependent relations. Coherence, in this view, is maintained through the continual regeneration of conditions that have no independent foundation. Building on this trajectory, the present section applies constraint closure to Meling’s (2021) account of NDA, showing how the shift from decoupled to precarious constraints models the experiential disclosure of groundlessness.

### Proposal: non-dual awareness as reduction of decoupled constraints

In Meling’s account, ‘phase-1 enaction’ refers to adaptive, norm-regulated sense-making: cognition organized by the system’s continual adjustment of environmental coupling in accordance with its viability norms. ‘Phase-2 enaction’, by contrast, marks a reorganization of this regulatory structure. The cognitive system ceases to evaluate experience through its own adaptive norms and becomes aware of the contingent, relational basis of its own organization. This experiential recognition of contingency corresponds to what Meling calls groundlessness—the realization that the apparent stability of self and world is itself dynamically enacted.

Viewed through the lens of constraint closure, this transformation can be described as a redistribution of causal influence across timescales. In ordinary cognition, higher-order, decoupled constraints—those governing prediction, normativity, and conceptual framing—stabilize the continual regeneration of faster, precarious relations. During NDA, these slower regulatory patterns relax. Feedback from the decoupled and double-decoupled orders weakens, allowing the system’s coherence to depend directly on the moment-to-moment regeneration of first-order relations. The result is not disorder but minimal closure: organization persisting without hierarchical modulation.

In this regime, viability is maintained through the same thermodynamic processes that underwrite all living organization—ongoing work that restores local asymmetries against dissipation—but now unfiltered by slower, predictive control. The system remains dynamically coherent even as the normative and representational scaffolds that usually stabilize that coherence subside. From the first-person standpoint, this manifests as dereification: the loosening of evaluative and narrative feedback loops that ordinarily sustain a sense of enduring self. Temporal continuity flattens, perception unfolds without interpretive distance, and cognition functions within a self-renewing immediacy. NDA is thus not a withdrawal from organization but awareness of organization as transient coordination.

Formally, let C₁, C₂, and C₃ denote precarious, decoupled, and double-decoupled constraints. In ordinary cognition, closure is maintained through the recursive modulation C₁ ↔ C₂ ↔ C₃. In NDA, the upward and downward couplings through C₂ and C₃ become attenuated, leaving coherence sustained primarily by the regeneration of C₁. Autonomy is preserved, but through immediacy rather than hierarchy—a system persisting through its most transient relations. In this sense, NDA embodies Nāgārjuna’s insight that emptiness (śūnyatā) is not voidness but dependent origination: coherence without intrinsic ground.

Finally, this interpretation distinguishes groundlessness from collapse. When constraint regeneration fails—as in burnout, derealization, or pathological dissociation—organization disintegrates. In NDA, regeneration continues at the fundamental level even as higher-order constraints relax. Coherence is thinned yet sufficient, achieved through the very instability that makes it possible. The system does not dissolve; it simplifies. NDA therefore illustrates, rather than originates, the principles of constraint closure—demonstrating how cognition can remain self-coherent through the regeneration of transient relations alone.

Importantly, the very notion of precarious constraints deepens the connection between constraint closure and Nāgārjuna’s analysis of emptiness. Precarious constraints are defined by their impermanence: they hold only by continuously renewing the conditions that sustain them. Their causal efficacy depends on ongoing regeneration, not on intrinsic stability or substance. In this sense, they instantiate the principle of śūnyatā—emptiness as conditionality without essence. What endures is not a self-subsistent structure but a pattern of dynamic co-dependence. To recognize the world as constituted by precarious constraints is, phenomenologically, to perceive the same groundlessness that Nāgārjuna describes philosophically and that Meling identifies experientially in NDA.

### Discussion and empirical implications

Reinterpreting NDA through constraint closure has both theoretical and empirical significance. Theoretically, it resolves a persistent tension within enactivism between autonomy and adaptivity. In standard process-closure accounts, adaptivity is necessary for sense-making and tightly coupled to autonomy in ‘phase-1 enaction’, yet it is not identical to autonomy. This leaves unclear how coherence persists when adaptive, norm-guided regulation subsides—as described in Meling’s ‘phase-2’ account of groundless awareness. Constraint closure supplies the missing mechanism: autonomy can persist wherever constraint regeneration continues, even when adaptive norms recede. NDA is therefore not a loss of autonomy but its minimal expression—a phase of organization sustained by precarious constraint dynamics alone.

Empirically, this model is broadly consistent with emerging neurophenomenological and dynamical-systems research on NDA. Conceptual and psychometric studies now treat NDA as a distinct dimension of consciousness, measurable at both trait and state levels (Hanley et al. 2018, Josipovic and Miskovic 2020, Josipovic 2021). Comparative typologies of focused-attention, open-monitoring, and deconstructive or non-dual practices indicate that advanced deconstructive training targets the dissolution of high-level self- and world-models rather than mere attentional stabilization (Travis and Shear 2010, Dahl et al. 2015, Lutz et al. 2015, Fox et al. 2016). At the neural level, fMRI work comparing non-dual awareness meditation with focused attention shows that anticorrelation between intrinsic/default-mode and extrinsic/task-positive systems becomes weaker during NDA, while within-network coherence is preserved—indicating a reconfiguration rather than a collapse of large-scale coupling (Josipovic et al. 2012, Fox et al. 2016). Electroencephalogram (EEG) and Magnetoencephalography (MEG) studies of non-dual or “selfless” states report increased power and more regular dynamics in slow alpha–theta bands, together with alterations in Default Mode Network (DMN)-related activity, consistent with relatively preserved or enhanced local synchrony alongside reduced long-range coordination (Berkovich-Ohana et al. 2013, Dor-Ziderman et al. 2013, Lee et al. 2018). Phenomenological analyses of self-boundary dissolution in meditation further show systematic trajectories in which the sense of location, agency, and first-person perspective attenuate while basic sensorimotor and interoceptive organization remain intact (Nave et al. 2021). These convergent empirical and phenomenological strands align with recent models that cast NDA as a graded shift in the explicitness of “consciousness-as-such” and in large-scale cortical topology (Josipovic 2021, Cooper et al. 2022).

From the standpoint of constraint-closure theory, this pattern can be interpreted as a temporary softening of second-order, decoupled constraints that normally coordinate predictive and executive hierarchies, while first-order, precarious constraints sustaining sensorimotor and interoceptive coherence remain closed. In neural-systems terms, NDA corresponds to a partial flattening of functional hierarchy: global integrative loops and long-range precision weighting become more labile, whereas local recurrent dynamics maintain viability. This interpretation dovetails with predictive-processing accounts of deconstructive meditation, which propose that advanced practice progressively relaxes high-level generative models and their precision (Laukkonen and Slagter 2021), and with free-energy-based models linking conscious integration to metastable, hierarchy-spanning dynamics under changing precision profiles (Friston 2010, Wiese and Friston 2021). Constraint-closure thus provides a principled vocabulary for explaining how experiential coherence can persist when higher-order predictive control relaxes but self-organizing local dynamics remain in place—a dynamical configuration increasingly associated, across converging empirical and phenomenological work, with NDA (Josipovic and Miskovic 2020, Josipovic 2021, Cooper et al. 2022).

Philosophically, this account unifies Nāgārjuna’s dependent origination, Juarrero’s relational causality, and Nave’s constraint closure within a single explanatory logic. All three reject intrinsic essence and treat stability as emergent from relational coordination. This dialogue follows the comparative methodology of Yuasa and Kasulis (1987), Chakrabarti and Weber (2015), and Thompson (2020), in which concepts from distinct traditions are placed in dialectical relation rather than assimilated to one framework. NDA is the experiential recognition of this logic: cognition aware of its own dynamic groundlessness.

This synthesis clarifies that “non-duality” does not signify metaphysical fusion but the absence of reification in the differentiation of self and world. Boundaries still function, but their contingency is evident—they are enacted, not given. NDA thus expresses epistemic transparency: direct awareness of relational dependence within cognition itself.

Finally, this integrative account bridges contemplative phenomenology and systems neuroscience. Both describe processes that sustain coherence without invoking intrinsic substance, exemplifying the dialectical convergence of Eastern and Western approaches to mind—the former through meditative insight into emptiness, the latter through dynamical models of self-organization. By articulating NDA as a minimal regime of constraint closure, this framework establishes a shared conceptual language for both domains and advances a formally tractable, cross-cultural science of mind.

## Conclusion

This article has argued that NDA can be understood as a reorganization of constraint dynamics that discloses the groundlessness of cognition itself. Tracing a conceptual progression from process closure to constraint causality and finally to constraint closure, the enactive framework evolves from describing autonomy as circular self-production to conceiving organization as the continuous regeneration of historically embedded relations. Each step removes a layer of substantialism: process closure risks presupposing invariant structures; constraint causality replaces intrinsic powers with relational conditions; and constraint closure shows that even these conditions are transient and self-producing. Cognition thus appears as coherence without foundation—a system sustained by the regeneration of its own contingent constraints.

Interpreting NDA through constraint closure clarifies how coherence can persist even when adaptive hierarchies relax. As decoupled constraints recede and precarious ones become dominant, cognitive organization remains dynamically sufficient despite its minimal form. Precarious constraints—defined by their instability and impermanence—embody the principle Nāgārjuna calls śūnyatā: dependence without intrinsic nature. The phenomenological experience of groundlessness thus reflects, at the organizational level, a system sustained by the continual regeneration of its own transient conditions. Far from signaling collapse, NDA reveals the resilience of autonomy under conditions of maximal openness.

At a broader level, this account participates in the project of cross-cultural cognitive science inaugurated by Varela et al. (1991) and expanded by Davis and Thompson (2013). That project envisions not the translation of Buddhist ideas into cognitive science but a reciprocal transformation between traditions. Following the methodological orientation of comparative philosophy (Kasulis 1987; Chakrabarti and Weber 2015, Thompson 2020), it treats Buddhist and scientific frameworks as partners in dialogue rather than as commensurable systems. As Thompson (2020) cautions, such dialogue must resist both “Buddhist naturalism” and the reduction of contemplative insight to neurobiological correlates. Its aim is clarification rather than assimilation—to reveal how each tradition illuminates the blind spots of the other.

In this spirit, the present analysis shows how Madhyamaka philosophy and constraint-based systems theory can be mutually clarifying. Nāgārjuna’s critique of intrinsic causation destabilizes that the substantialist assumptions often implicit in Western models of mind, while Juarrero’s and Nave’s relational theories of constraint render that critique formally intelligible within contemporary dynamical-systems science. Constraint closure thereby functions not as a metaphysical importation but as a conceptual bridge: it formalizes the shared insight that coherence arises only through interdependent regeneration.

NDA provides a uniquely tractable context for testing this bridge. It represents a deliberately cultivated condition in which conceptual mediation and self-referential regulation are minimized, allowing neural and phenomenological dynamics to converge on the same principle of groundless interdependence. Empirical findings from contemplative neuroscience already suggest such patterns: reductions in large-scale synchronization alongside locally stabilized coherence (Josipovic 2014, Timmermann et al. 2023). These results imply that the experience of emptiness may have an identifiable organizational correlate—the redistribution of constraint hierarchies that sustains coherence through impermanence.

Ultimately, this work contributes to both enactivism and comparative philosophy. For cognitive science, it reframes autonomy as the ongoing regeneration of relational constraints rather than the preservation of fixed networks. For philosophy, it demonstrates that Nāgārjuna’s analysis of dependent origination can be articulated within a rigorous scientific vocabulary without collapsing into reductive naturalism. The convergence of Madhyamaka, constraint theory, and enactivism does not yield a single unified framework but an interdisciplinary clearing—a space where the study of mind and the realization of emptiness mutually illuminate one another. In this sense, the dialogue that Varela envisioned between phenomenology, Buddhism, and cognitive science remains not a completed synthesis but an evolving practice of reciprocal transformation.

## Data Availability

No new data were generated or analyzed in support of this research.
